# Laparoscopic total gastrectomy using the transorally inserted anvil (OrVil™): a preliminary, single institution experience

**DOI:** 10.1186/2193-1801-3-434

**Published:** 2014-08-14

**Authors:** Fabio Cianchi, Giuseppe Macrì, Giampiero Indennitate, Beatrice Mallardi, Giacomo Trallori, Maria Rosa Biagini, Benedetta Badii, Fabio Staderini, Giuliano Perigli

**Affiliations:** Department of Surgery and Translational Medicine, University of Florence, Florence, Italy; Department of Experimental and Clinical Biomedical Sciences, University of Florence, Florence, Italy; IFCA, Florence, Italy; ISPO, Florence, Italy; Endocrine and Minimally Invasive Surgery, Azienda Ospedaliero-Universitaria Careggi, Center of Oncologic Minimally Invasive Surgery (COMIS), Department of Surgery and Translational Medicine, University of Florence, Largo Brambilla 3, 50134 Florence, Italy

**Keywords:** Gastric cancer, Laparoscopic total gastrectomy, Esophagojejunal anastomosis, Intracorporeal circular stapling

## Abstract

Laparoscopic total gastrectomy (LTG) is not a commonly performed procedure due to the difficulty associated with surgical reconstruction. We present our preliminary results after intracorporeal circular stapling esophagojejunostomy using the newly developed transorally inserted anvil (OrVil™, Covidien, MA, USA). Between 2008 and June 2013, 51 patients underwent laparoscopic gastrectomy with D2 lymph node dissection for gastric cancer. A total of 12 patients underwent LTG: of these, 5 received an intracorporeal linear side-to-side esophagojejunal anastomosis and the remaining 7 underwent intracorporeal circular stapling esophagojejunostomy using the OrVil™ system. Short-term outcomes were compared between the two groups. There were no intraoperative complications or conversions to open surgery in any patients. The mean operative time was significantly shorter in the OrVil™ than in the side-to-side group (261.4 ± 12.0 vs 333.0 ± 15.0 minutes, respectively, p = 0.005). Postoperative fluorography revealed no anastomosis leakage or stenosis in either groups. All patients resumed an oral liquid diet on postoperative day 5 and the mean postoperative hospital stay was 9 days. Intracorporeal circular stapling esophagojejunostomy using the OrVil™ system is technically feasible and safe in LTG. This technique may be considered a simple and time-saving alternative to the side-to-side linear esophagojejunostomy.

## Background

Laparoscopic gastrectomy has become an acceptable alternative approach to open surgery in the management of early and more recently, advanced gastric cancer especially in Eastern countries (Ohtani et al. 2010; Qiu et al. 2013). A growing number of studies have demonstrated the feasibility and oncologic efficacy of laparoscopic distal gastrectomy which also provides all the well known clinical advantages of minimally invasive surgery (Jiang et al. 2013; Ding et al. 2012). On the contrary, laparoscopic total gastrectomy (LTG) is still a much less common operative procedure. This is basically due to the more difficult technique of LTG, in particular when an intracorporeal esophagojejunal anastomosis is performed. Some authors have proposed an extracorporeal esophagojejunostomy through minilaparotomy (Kim et al. 2008; Usui et al. 2005; Mochiki et al. 2008; Shim et al. 2013), but this method does not always safely allow insertion of an anvil, due to the narrow operating window especially in obese patients. Although other studies have described some modified techniques of esophagojejunostomy after LTG in a attempt to overcome these technical problems, an optimal and standard laparoscopic procedure has yet to be established.

In the present study, we present our preliminary results after intracorporeal circular stapling esophagojejunostomy using the newly developed transorally inserted anvil (OrVil™, Covidien, Mansfield, MA, USA).

## Results

A total of 12 patients underwent LTG with Roux-en-Y reconstruction. The first 5 patients (3 men; 2 women; mean age 66.0 ± 6.8) received an intracorporeal linear side-to-side esophagojejunal anastomosis. The remaining 7 patients (6 men; 1 woman; mean age 74.1 ± 3.0) underwent intracorporeal circular stapling esophagojejunostomy using the OrVil™ system. None of the patients had a past history of abdominal surgery. There were no significant differences in BMI and co-morbidities between the side-to-side and OrVil™ patient groups (26.5 ± 0.9 vs 25.5 ± 1.1 kg/m^2^ and 40.0% vs 57.1%, respectively, p = NS).

All patients successfully underwent LTG without intraoperative complications or conversion to open surgery. The mean operating time in the OrVil™ group was significantly shorter than in the side-to-side esophagojejunal anastomosis group (261.4 ± 12.0 vs 333.0 ± 15.0 minutes, respectively, p = 0.005). The estimated blood loss did not significantly differ between the two groups (126.7 ± 12.7 ml in the side-to-side group vs 115 ± 9.6 ml in the OrVil™ group, p = NS). There were no postoperative surgery-related complications in the two groups. Postoperative fluorography was performed on postoperative day 5 in all patients and demonstrated no leakage or stenosis of esophagojejunal anastomosis. All patients resumed an oral liquid diet on postoperative day 5 and the overall mean hospital stay was 9.2 ± 0.9 days. All patients underwent endoscopic control 6 months after surgery: no anastomotic stricture occurred in any patients.

Pathological examination revealed 1 stage IIA, 3 stage IIIB and 1 stage IIIC tumors in the side-to-side esophagojejunal anastomosis group. In the OrVil™ group, there were 1 stage IA, 1 stage IB, 1 stage IIA, 2 stage IIIA and 2 stage IIIB tumors. All resected specimens had tumor-free resection margins and the mean number of harvested lymph nodes did not significantly differ between the OrVil™ (32.4 ± 4.2) and the side-to-side (29 ± 2.4) patient group. After a median follow-up of 15.5 months (range 7–34), no patient within the two groups developed local or anastomotic tumor recurrence.

Demographic characteristics and surgical outcomes of patients within the two study groups are summarized in Table 1.

**Table 1:** **Comparison of demographic characteristics and surgical outcomes between the side-to-side esophagojejunal anastomosis and the OrVil**™ **group**

	Side-to-side esophagojejunal anastomosis N. 5	Esophagojejunal anastomosis with OrVil™ N. 7	***P***value
**Age** (mean ± SEM)	66 ± 6.8	74.1 ± 3.0	NS
**Gender** (M:F)	3:2	6:1	NS
**BMI** (kg/m^2^) (mean ± SEM)	26.5 ± 0.9	25.5 ± 1.1	NS
**Previous abdominal surgery** (total)	0	0	NS
**Comorbidities** (total)	2 (40%)	4 (57.1%)	NS
Hypertension	1	2	
Diabetes mellitus	1	0	
Heart diseases	0	1	
Chronic lung diseases	0	1	
**Mean operative time** (min) (mean ± SEM)	333.0 ± 15.0	261.4 ± 12.0	0.005
**Blood loss** (ml) (mean ± SEM)	126.7 ± 12.7	115 ± 9.6	NS
**Surgery-related complications** (total)	0	0	NS
**N. of retrieved lymph nodes after D2 dissection** (mean ± SEM)	29 ± 2.4	32.4 ± 4.2	NS

## Discussion

The advantages of laparoscopic distal gastrectomy in the treatment of both early and advanced gastric cancer have been well investigated. On the other hand, LTG has not become as popular as distal gastrectomy due to a low incidence of upper gastric cancer necessitating total gastrectomy and the technical difficulty of LTG, especially for the reconstruction procedure (Kim et al. 2008; Usui et al. 2005; Mochiki et al. 2008; Shim et al. 2013; Cianchi et al. 2013; Lee et al. 2013). Although various types of esophagojejunostomy after LTG have been proposed, a standard and optimal procedure has not yet been established. One commonly used method is extracorporeal esophagojejunostomy which is similar to conventional open surgery (Kim et al. 2008; Usui et al. 2005; Mochiki et al. 2008; Shim et al. 2013; Lee et al. 2013). Although this technique is familiar to most surgeons, it is often difficult to complete esophagojejunal anastomosis through minilaparotomy because of the narrow operating window for anvil insertion and application of instruments, especially in obese patients. It can be associated with a high risk of bleeding and temporary anastomosis ischemia due to excessive traction of the mesentery. Moreover, carrying out a larger laparotomy would make it similar to conventional open surgery with loss of the well-documented advantages of minimally invasive surgery.

The alternative method is intracorporeal esophagojejunostomy. It can be performed by using either a circular or an endoscopic linear stapler. In total laparoscopic procedures using a conventional circular stapling device, the insertion of the anvil head into the distal esophagus is the most technically challenging and stressful step. To facilitate this step, various “purse-string” procedures have been proposed: Takiguchi et al. (2005) developed a laparoscopic purse-string suture technique using a semiautomatic suturing device, namely the Endostitch (Covidien), while Usui et al. (2008) developed an endoscopic purse-string instrument, called the Endo-PSI (Hope Electronics, Chiba, Japan). Inaba et al. (2010) introduced the “overlapped” esophagojejunostomy, especially designed to reduce the complexity of anastomosis with a circular stapler. Altogether these techniques appear to be technically demanding and time consuming (Shim et al. 2013) and carry a potential risk of increasing the incidence of postoperative complications.

Side-to-side linear esophagojejunostomy has been used by various authors with excellent results. An Italian multicenter study (Bracale et al. 2010) has shown that this approach is safe and feasible, with a low incidence rate of anastomotic leakage (6%) and stenosis (3%). These authors thus concluded that this procedure represents the best choice for reconstruction of the digestive tract after LTG. In the first 5 patients we performed this technique and, although we cannot provide definitive results because of the low number of patients, we did not register any intra- or post-operative complications. Nevertheless, side-to-side intracorporeal esophagojejunostomy is a time-consuming procedure given the need to close the entry hole through which the stapler has been inserted. Our average operative time was quite a bit longer than that reported by the Italian study (Bracale et al. 2010) (333 vs 249 minutes) and this was likely due to the fact that we used interrupted stitches with extracorporeal knot-tying to close the entry hole. Moreover, we found it difficult to introduce the linear stapler into the esophagus due to the risk of esophageal injury from the dissection of the space between the muscular layers and mucosa. For these reasons, we adopted the OrVil™ technique in the last 7 procedures. In this method, the transoral placement of the anvil simplified the anvil insertion procedure and there is no need to perform purse-string suturing. Furthermore, the intracorporeal stapling technique is not technically demanding due the use of a circular stapler and this, in our experience, significantly reduced the operative time in comparison with side-to-side linear esophagojejunostomy. We have successfully performed this procedure without conversion to open surgery or perioperative complications (e.g., leakage or stenosis) associated with the anastomosis.

Our results are in line with those reported by other authors. Kunisaki et al. (2011) used the OrVil™ technique after LTG for cancer in 30 patients and demonstrated a significant reduction of operative time and blood loss in comparison with a group of patients who received a Roux-en-Y esophagojejunostomy reconstruction through a mini-laparotomy. Other studies report satisfactory outcomes with the OrVil™ system for both esophagojejunostomy after LTG (Jeong and Park 2009; Liao et al. 2013) and gastrojejunostomy in laparoscopic Roux-en-Y gastric bypass (Chavarriaga et al. 2010). Recently, LaFemina et al. (2013) reported the first American experience with the use of the OrVil™ system in both open and laparoscopic total gastrectomy. The characteristics of their 17 patients who underwent LTG, in particular the median value of BMI (27.1 kg/m^2^), were comparable with those of our patients. These authors concluded that the OrVil™ technique can minimize the technical difficulties in creating intracorporeal stapled circular esophagojejunostomy, with an incidence of complications comparable with that occurring after previously proposed procedures.

The earliest study on OrVil™ after LTG (Jeong and Park 2009) encountered postoperative infection because the contaminated OrVil™ tube enters the abdominal cavity during the procedure. For this reason, some authors have recommended that the patients perform oral gargling with hexamidine solution before surgery and abundant abdominal irrigation just after anvil insertion. In our series, we did not observe postoperative infection, although we acknowledge that abdominal irrigation at the end of the operation may be sufficient to avoid abdominal infection.

## Conclusions

Although our study is limited by the small sample size, we demonstrate that an intracorporeal circular stapling esophagojejunostomy during LTG can be performed safely and effectively using the OrVil™ system. This technique may be considered a simple and time-saving alternative to the side-to-side linear esophagojejunostomy.

## Methods

From June 2008 to June 2013, 51 patients underwent laparoscopic gastrectomy with D2 lymph node dissection for gastric cancer at the Center of Oncologic Minimally Invasive Surgery, University of Florence, Italy. A total of 12 patients underwent LTG: of these, 5 received a Roux-en-Y intracorporeal linear side-to-side esophagojejunal anastomosis and the remaining 7 underwent intracorporeal circular stapling esophagojejunostomy using the OrVil™ system. All LTGs were performed by a single surgeon (F.C.) after an adequate learning curve with 31 laparoscopic distal gastrectomies. All patients underwent diagnostic and preoperative staging work-up according to a standard protocol which includes upper digestive endoscopy with gastric biopsy and computed tomography of the abdomen and chest. Patients with distant metastases, para-aortic lymph node involvement and/or pre- or intraoperative diagnosis of T4 lesions (i.e., local invasion of other organs, including spleen, pancreas or peritoneum), were excluded from the study. All patients had been thoroughly informed about the study and gave written consent for the investigation in accordance with the ethical guidelines of our University.

The characteristics of patients, such as age, gender, body mass index (BMI), history of abdominal surgery, co-morbidities and surgical outcomes (operative time, blood loss, intra- and postoperative complications, mortality, time-to-first oral intake, postoperative hospitalization and pathological results) were analyzed.

### Surgical technique

Under general anesthesia, the patient was placed in supine, reverse Trendelenburg position with legs abducted. The surgeon stood between the legs of the patient. Four trocars were used. One 10–12 mm trocar for the laparoscope was inserted into the umbilicus. Another 10–12 mm trocar was inserted in left midclavicular line 2 cm above the umbilicus as a major hand port. Two other 5 mm trocars were inserted in the right midclavicular line 2 cm above the umbilicus and in the midline just below the xiphoid process. First, a routine exploration of the abdominal cavity was performed. D2 lymphadenectomy and gastric dissection were performed as described previously (Cianchi et al. 2013).

Side-to-side esophagojejunal anastomosis. A Roux-en-Y intracorporeal linear side-to-side esophagojejunal anastomosis was performed in the first 5 procedures. After the dissection step, the entire stomach was mobilized but not yet sectioned from the esophagus. To make a Roux limb, the jejunum was transected from 20 to 30 cm below the Treitz’s ligament using a linear stapler. Two access openings were created and the forks of a 45-mm cartridge linear stapler were inserted into the esophagus and the jejunum to create a side-to-side anastomosis. The entry hole was closed with interrupted stitches. The stomach was then completely sectioned from the esophagus using a linear stapler. In these patients, the specimen was pulled out through the umbilical port and the jejunum-jejunostomy was constructed with a linear stapler via this minilaparotomy.

**Figure 1:**
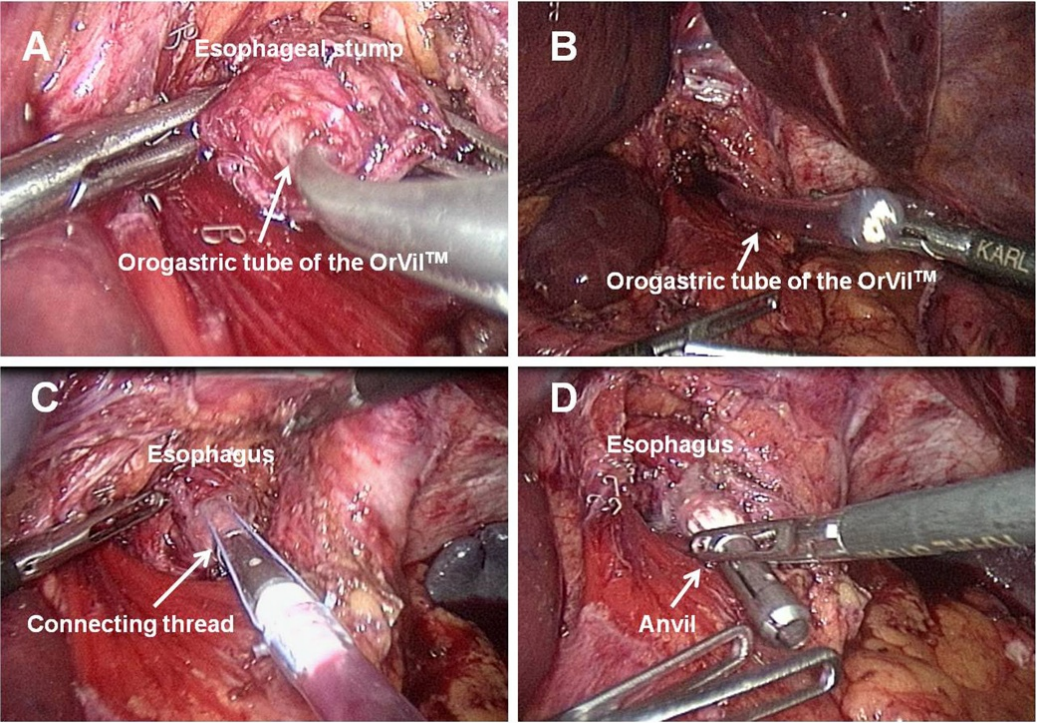
**Intracorporeal anvil insertion using the OrVil™ system. A**. As the OrVil™ tube reaches the esophageal stump, a small hole is created on the stump. **B**. The tube is pulled out through the hole, until the anvil reaches the esophageal stump. **C**. The tube is disconnected from the anvil by cutting the connecting thread and then removed from the abdominal cavity. **D**. Laparoscopic view after completion of the transoral anvil insertion.

**Figure 2:**
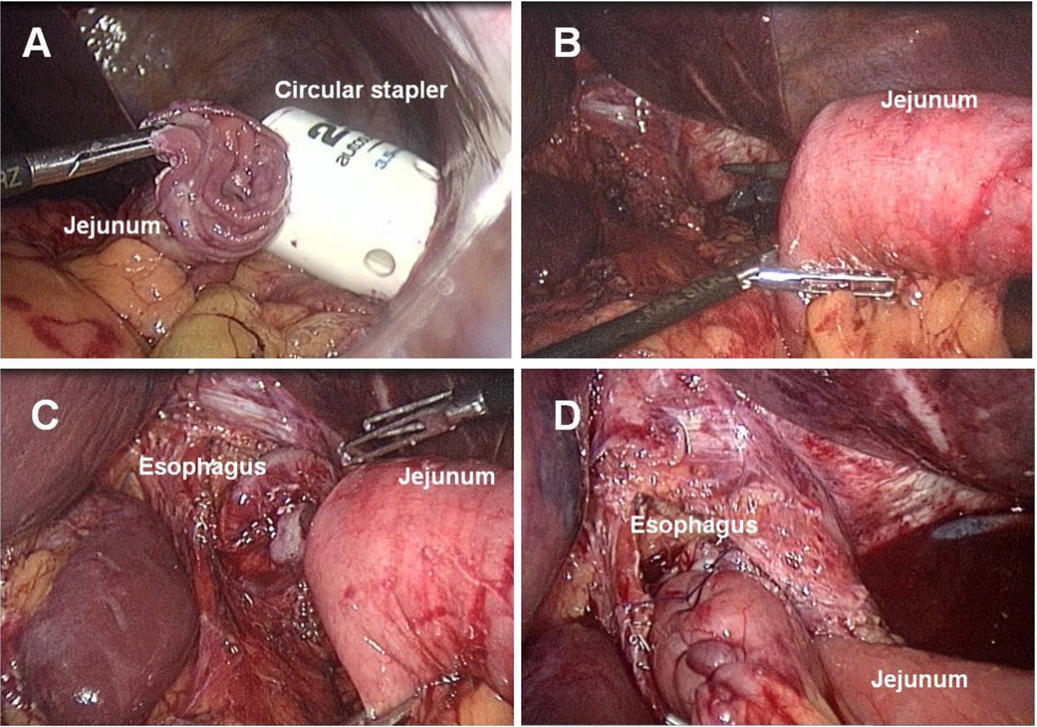
**Intracorporeal circular stapling technique. A**. A circular stapler is introduced into the abdomen through the left midclavicular line port which is extended to a length of 4–6 cm. **B**. After pneumoperitoneum is re-established, the circular stapler is inserted into the jejunum. **C**. Double-stapling esophagojejeunostomy is performed under direct laparoscopic view. **D**. Laparoscopic view after completion of the anastomosis.

Intracorporeal circular esophagojejunostomy. In the last 7 patients, we used the newly developed transorally inserted anvil (OrVil™, Covidien) and a circular stapler (EEA25, Covidien) to perform an intracorporeal circular esophagojejunostomy. The OrVil™ system includes an anvil with an orogastric tube attached to the central rod by a connecting thread. After the dissection step, the esophagus was transected with a 45-mm linear stapler. The orogastric tube was introduced by the anesthetist into the esophagus through the mouth and pushed down to the esophageal section line. After an incision is made at this level, the tube was retrieved until the anvil reached the esophageal stump (Figure 1A and B), at which time the tube was disconnected from the anvil by cutting the connecting thread and removed from the abdominal cavity (Figure 1C and D). Abdominal irrigation around the esophageal stump was performed to prevent postoperative abdominal infection. The jejunal loop for subsequent Roux-en-Y reconstruction was transected with a linear stapler as previously described. The left midclavicular line port was extended to a length of 4–6 cm and the circular stapler was introduced into the abdominal cavity through this minilaparotomy which was partially closed to re-establish the pneumoperitoneum (Figure 2A). The stapler was inserted into the jejunal limb and connected with the anvil to perform anastomosis under direct laparoscopic view (Figure 2B,C and D). Finally, the jejunal stump was closed with an endoscopic linear stapler. The specimen was pulled out of the peritoneal cavity through the minilaparotomy and the jejunum-jejunostomy was constructed with a linear stapler through this access.

A silicon drainage tube was placed around the esophagojejunal anastomosis in all patients.

### Statistical analysis

Categorical variables within the two study groups were compared using the chi-square test and Fisher’s exact test. Quantitative variables were summarized by mean ± SEM. Groups were compared using the Mann–Whitney test. All of the Ps resulted from the use of two-sided statistical tests; a P < 0.05 was considered statistically significant.

## Abbreviations

LTG: Laparoscopic total gastrectomy
BMI: Body mass index.
